# Unexplained interannual oscillations of cyanobacterial blooms in the Baltic Sea

**DOI:** 10.1038/s41598-018-24829-7

**Published:** 2018-04-23

**Authors:** Mati Kahru, Ragnar Elmgren, Emanuele Di Lorenzo, Oleg Savchuk

**Affiliations:** 1 Scripps Institution of Oceanography, University of California San Diego, La Jolla, CA 92093-0218 USA; 2 Department of Ecology, Environment and Plant Sciences, Stockholm University, Stockholm, Sweden; 3 School of Earth and Atmospheric Sciences, Georgia Institute of Technology, Atlanta, GA 30332 USA; 4 Baltic Nest Institute, Stockholm University, Stockholm, Sweden

## Abstract

Population oscillations in multi-species or even single species systems are well-known but have rarely been detected at the lower trophic levels in marine systems. Nitrogen fixing cyanobacteria are a major component of the Baltic Sea ecosystem and sometimes form huge surface accumulations covering most of the sea surface. By analysing a satellite-derived 39-year (1979–2017) data archive of surface cyanobacteria concentrations we have found evidence of strikingly regular interannual oscillations in cyanobacteria concentrations in the northern Baltic Sea. These oscillations have a period of ~3 years with a high-concentration year generally followed by one or two low-concentration years. Changes in abiotic factors known to influence the growth and survival of cyanobacteria could not provide an explanation for the oscillations. We therefore assume that these oscillations are intrinsic to the marine system, caused by an unknown, probably mainly biological mechanism that may be triggered by a combination of environmental factors. Interactions between different life cycle stages of cyanobacteria as well as between predator-prey or host-parasite are possible candidates for causing the oscillations.

## Introduction

**Figure 1:**
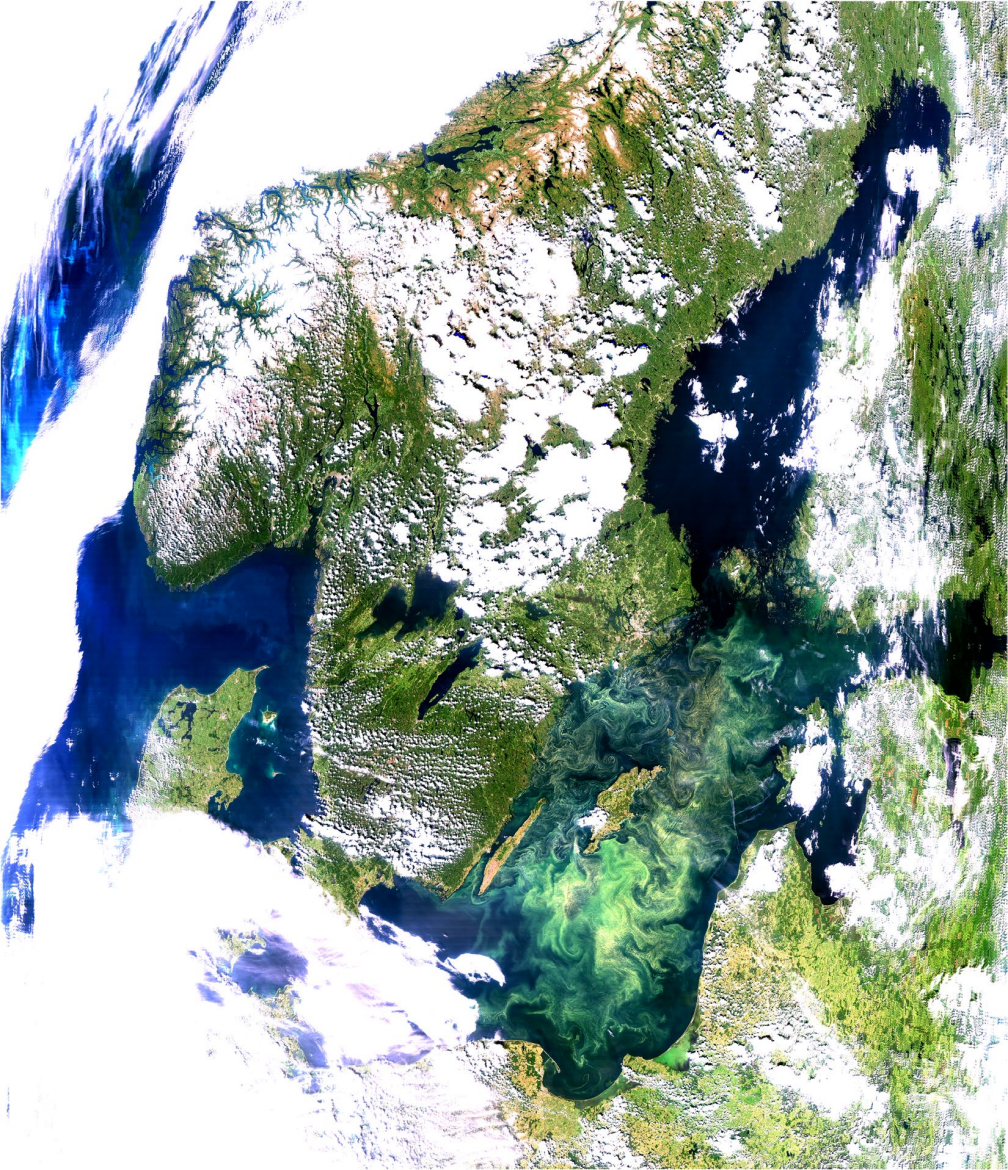
Satellite view of the Baltic Sea on 8-July-2005. Cyanobacteria accumulations are visible as bright squiggly structures covering most of the Baltic Proper. Data from NASA’s MODIS-Aqua sensor acquired from the Level-1 and Atmosphere Archive & Distribution. System (LAADS) Distributed Active Archive Center (https://ladsweb.nascom.nasa.gov/). The quasi-true colour image was produced by M. Kahru using WIM/WAM software described in http://www.wimsoft.com/Course/2_Level_1B/Exercises_modis_250m.pdf.

Population oscillations produced by predator-prey or host-parasite interactions^1–3^ or even in single species^4^ are well-known but have rarely been detected at the lower trophic levels in marine systems. Nitrogen fixing cyanobacteria have been an important component of the Baltic Sea ecosystem for millennia^5–8^. During the summer they often form massive surface blooms^9^, in some years covering as much as 200,000 km^2^ (Fig. 1). Their ability to fix nitrogen makes cyanobacteria an important driver in the nitrogen cycle by stimulating overall primary production and hence contributing to anoxic conditions at the bottom of the sea^6,8,10^. The propensity of the co-dominant genus *Nodularia* to form near-surface accumulations makes it feasible to map cyanobacteria accumulations using satellite sensors^11^. As satellite detection of cyanobacteria accumulations is limited to periods of clear skies and available satellite overpasses, an index called frequency of cyanobacteria accumulations^9,12^ (FCA) normalizes the number of detections to the number of observations. Time-series of FCA in the Baltic Sea now span 39 years (1979–2017) and show both decadal-scale and interannual variability. By analysing the data archive of detrended surface cyanobacteria bloom frequency, we found evidence of strikingly regular interannual fluctuations in the northern Baltic Sea. These oscillations have a period of ~3 years with a high-concentration year generally followed by one or two low-concentration years. We are not aware of similar 3-year oscillations in the Baltic or any other marine systems in the World.

## Results

**Figure 2:**
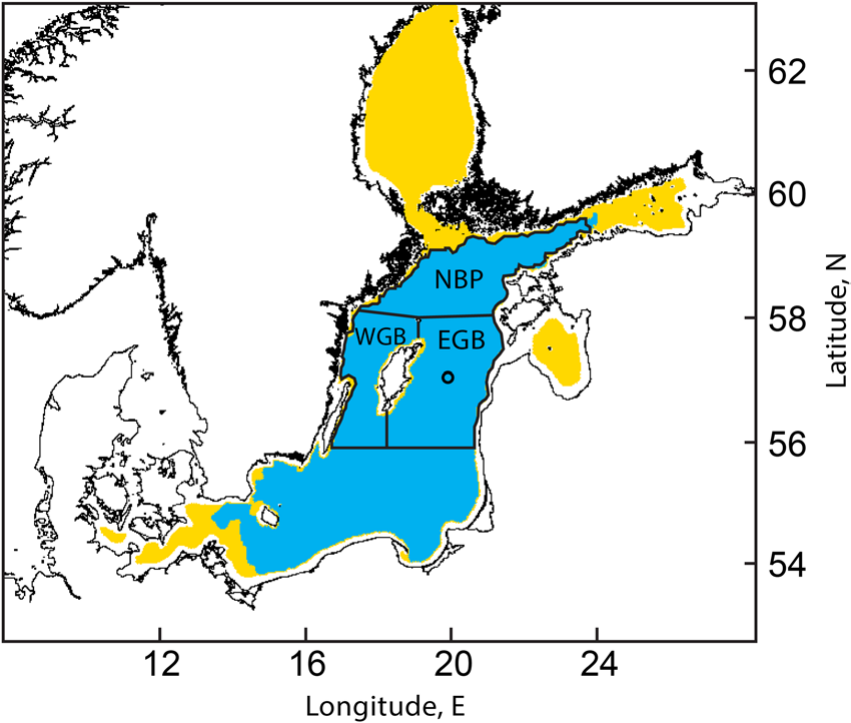
Map of the study area showing the central Baltic Sea (blue) that excludes shallow coastal areas (<15 m) and the gulfs. The sub-basins of Northern Baltic Proper (NBP), Western Gotland basin (WGB) and Eastern Gotland Basin (EGB) are shown. The small circle in EGB shows the location of the Baltic monitoring station BY15. Map produced using WIM/WAM software http://www.wimsoft.com.

As near-coast turbidity and resuspended sediments from the bottom can interfere with our method of cyanobacteria detection, we evaluate FCA in central parts of the Baltic Sea deeper than 15 m (Fig. 2). FCA time series described in Kahru & Elmgren^9^ was extended to 2017 by using data from MODIS-Aqua, MODIS-Terra and VIIRS satellite sensors. Trends and longterm changes in the time series were removed with the first difference method (y_t_ = x_t_ − x_t−1_).

**Figure 3:**
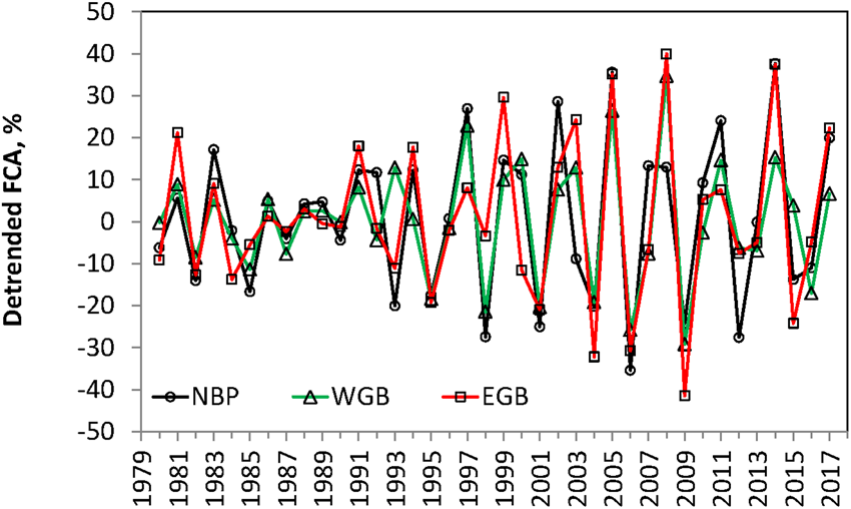
Time series of the detrended (with the first difference transformation) frequency of cyanobacteria accumulations (FCA) for three sub-areas of the northern Baltic Sea: Northern Baltic Proper (NBP), Western Gotland Basin (WGB) and Eastern Gotland Basin (EBP) (for locations see Fig. 2).

In the northern basins of the Baltic Sea Proper (Fig. 2) the interannual changes are strikingly regular with each high year soon (generally in 1–2 years) followed by a low year and vice versa (Fig. 3). Peaks and troughs observed in adjacent spatial domains are highly synchronous with only minor differences. During the 39 years of observations we count 14 peaks, making the average period 2.8 years long. The amplitude of the oscillations was particularly high during the 2005–2015 period.

Cyanobacteria blooms are known to be stimulated by a low nitrogen to phosphorus ratio and high water temperature^13–15^. Their accumulation in near surface waters is enhanced by high sea-surface temperature, high incoming radiation and low wind speed^11,16^. We therefore examined a set of 29 environmental variables (see Supplementary Information, Table 1) related to major factors influencing the growth and survival of cyanobacteria blooms such as sea-surface temperature (SST), salinity, incoming radiation, winds, concentrations of major nutrients and the extent of hypoxic areas in the Baltic Sea. For example, we used the number of days and sums of SSTs with temperature higher than 14 °C and 17 °C, respectively, as those variables, in addition to the average SST can influence the dynamics of cyanobacteria blooms. We also used the average sunshine duration and shortwave irradiance at the surface as well as wind velocities as those variables are expected to influence surface manifestations of the blooms. Partial least squares (PLS) regressions were used to find combinations of variables that can explain the observed oscillations in FCA while the strength of the relationships was evaluated with the coefficient of determination (r^2^). It appears that while the decadal scale changes, particularly when averaged over the whole central Baltic Sea, are correlated with environmental variables and can be predicted with reasonable accuracy (r^2^ = 0.58) using combinations of various environmental variables, the year-to-year oscillations of FCA, particularly regular in the northern Baltic, could not be predicted with any significant accuracy using any of the studied environmental variables or their combinations (r^2^ < 0.15). When evaluated individually, none of the included environmental variables had a significant correlation with the detrended FCA in the northern Baltic at the 95% confidence level. Only two variables - sums of daily SSTs above 14 °C and 17 °C, had positive correlations at 90% confidence level (p < 0.1).

## Discussion and Conclusions

While we cannot find any abiotic environmental factors responsible for causing the observed oscillations in cyanobacteria blooms we can envision several possible biological mechanisms. Interactions between different life-cycle stages of cyanobacteria, e.g. the pelagic growing cells and the benthic resting cells, can influence the seasonal cycle and the year-to-year fluctuations^17^. However, higher abundance of vegetative cells would lead to more resting cells and therefore to a higher “inoculum” which would cause clusters of years with either high or low abundance and not the observed up-down fluctuations. Relatively little is known about the cyanobacteria-zooplankton interactions that can also cause cyanobacteria population changes. While the filamentous toxin-producing Baltic cyanobacteria are consumed as food by zooplankton, they are not a preferred food and seem to be consumed mostly when other forms of phytoplankton are in low supply^18–20^. Based on current understanding, it is therefore difficult to imagine a tight predator-prey relationship between zooplankton and the filamentous cyanobacteria that is able to produce the observed oscillations. Possible interactions between cyanobacteria and viruses or cyanobacteria and some biogeochemical compounds left in the environment after a bloom are even less known and the prolonged survival of such effects until the next summer are difficult to imagine. In conclusion, we currently lack a plausible explanation for the strong interannual oscillations in the frequency of cyanobacteria surface accumulations observed in the Baltic Sea, but consider a mainly biological mechanism likely.

## Methods

### Frequency of cyanobacteria accumulations (FCA)

As satellite detection of cyanobacteria accumulations is limited to periods of clear skies and available satellite overpasses, FCA normalizes the number of detections to the number of observations. Calculated for each pixel, FCA^9^ is the ratio of the number of days when cyanobacteria accumulations were detected to the total number of days with unobstructed satellite views of the sea surface. As cyanobacteria accumulations in the Baltic Sea occur almost exclusively during the summer months, FCA was averaged over the 2-month period of July–August.

### Statistical significance of the oscillations

**Figure 4:**
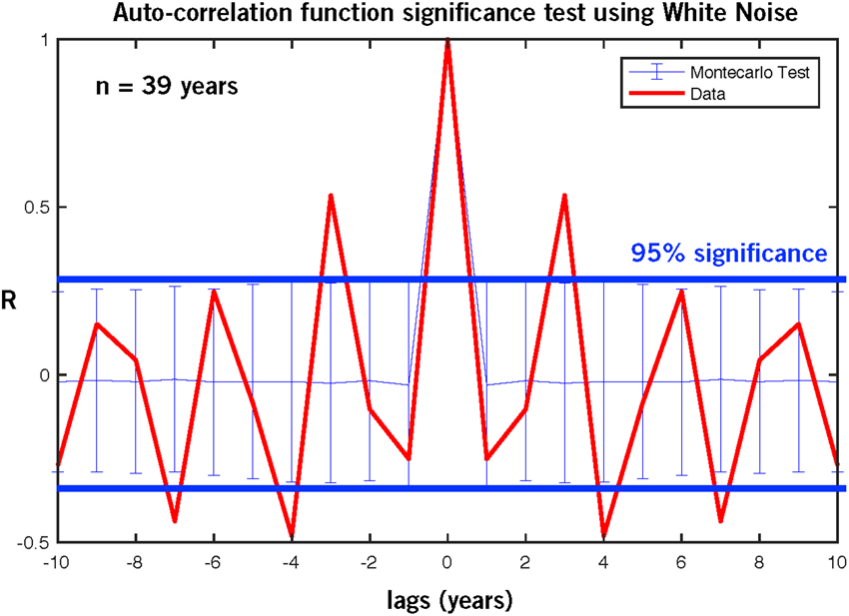
Test of the statitistical significance of the oscillations using simulated autoregressive series.

We examined both the full time series (1979–2017, n = 39) and the part after 1995 (n = 23), with the highest frequency of satellite coverage. The sample autocorrelation function (XCF) derived from the FCA sequences after 1995 exhibited a clear set of peaks at different lags, i.e. oscillatory nature. Using the entire length of the FCA sequences did not change the character of the auto-correlation function. Specifically, the XCFs show a recurrent peak on timescales of ~3 years (r > 0.5) suggesting a dominant periodicity in the data. To determine if the oscillations in the auto-correlation functions are significant, given the relatively short length of the time-series, we developed a Monte Carlo test where we computed the XCF of 10,000 realizations of white noise that have equal length as the original data (e.g. number of samples), and used the XCFs to compute the 95% confidence level associated with each lag in the XCF. We found that (Fig. 4) the peak at lag 3 years in the XCF was significant above the 95% level both for the full time series and for the samples after 1995.

Additional information on data and methods is available in the Supplementary Information in the online version of this paper.

### Data availability

The authors declare that the main data supporting the findings of this study are in the online Supplement of the paper and also available from the author’s website http://www.wimsoft.com/BalticCyano/BalticCyanoOscillation.htm. Additional data are available from the corresponding author upon request.

## Electronic supplementary material

Supplementary Information
