# Supplementary material for: Unexplained interannual oscillations of cyanobacterial blooms in the Baltic Sea
Source: Sci Rep. 2018 Apr 23;8:6365. doi: 10.1038/s41598-018-24829-7 (PMC5913306; doi:10.1038/s41598-018-24829-7)
Supplement: Supplementary file 1 — Supplementary Information [file 41598_2018_24829_MOESM1_ESM.docx]

Unexplained interannual oscillations of cyanobacterial blooms in the Baltic Sea

Mati Kahru^1*^, Ragnar Elmgren^2^, Emanuele Di Lorenzo^3^ and Oleg Savchuk^4^

^1^Scripps Institution of Oceanography, University of California San Diego, La Jolla, CA 92093-0218, USA

^2^Department of Ecology, Environment and Plant Sciences, Stockholm University, Stockholm, Sweden

^3^School of Earth and Atmospheric Sciences, Georgia Institute of Technology, Atlanta, GA 30332, USA

^4^Baltic Nest Institute, Stockholm University, Stockholm, Sweden

^*^email: mkahru@ucsd.edu

# Supporting Material

**Additional methods and materials**

**Sea-surface temperature (SST).** Time series of the sea-surface temperature (SST) were obtained from optimally interpolated global blended AVHRR temperatures (Reynolds et al. 2007) (<https://podaac.jpl.nasa.gov/dataset/AVHRR_OI-NCEI-L4-GLOB-v2.0>). The following five variables were computed: mean SST, the number of days and the sum of temperatures, respectively, over 14˚C and over 17˚C. All these variables were calculated pixel-wise and then averaged for the July-August period.

**Surface shortwave radiation.** Sunshine duration (SDU), surface incoming shortwave direct irradiance (SID) and surface incoming shortwave irradiance (SIS) were obtained from the SARAH-2 dataset based on geostationary Meteosat data (Kothe et al. 2017; Mueller et al. 2012, 2015) and downloaded from the Satellite Application Facility on Climate Monitoring (CM SAF, <http://www.cmsaf.eu/EN/Products/AvailableProducts/Dataset/Dataset_node.html>).

**Near-surface winds.** Time series of vector sea surface winds blended from multiple satellites (Zhang et al. 2006) were acquired from NOAA's National Climatic Data Center, via their website <https://www.ncdc.noaa.gov/data-access/marineocean-data/blended-global/blended-sea-winds>. The eastward and northward components were averaged over the July-August period from daily datasets.

**Biogeochemical data.** Time series of basin-wide total amounts of nutrients and the area of surface covering waters containing no more than 2 mL L^-1^ of oxygen (hypoxic area, HA) were estimated from 3D gridded fields annually aggregated and averaged with the Data Assimilation System (<http://nest.su.se/das/>) from observations over the entire Baltic Proper (Savchuk 2010). Time series of marine observations in the layer 0-15 m in the area of international monitoring station BY15, in the northern, and the entire Baltic Proper were pooled together and averaged within consecutive half-month intervals over 1979-2016 with the Decision Support System Baltic Nest (<http://www.balticnest.org>). All the data were acquired from the Baltic Environmental Database (BED) and a system of distributed databases maintained in Denmark, Finland, Germany, and Sweden (<http://nest.su.se/bed/>).

**Partial least squares linear regression** (PLS) as implemented in NMath numerical libraries (<http://www.centerspace.net>) was used to search for environmental variables that could explain (predict) the time series of FCA. The list of environmental variables used in PLS is shown in Supplementary Table 1. As time series of most environmental variables start in 1987 and miss data for 2017, regressions were performed for the 1987-2016 period.

**Supplementary Table S1**. Environmental variables used in PLS to predict FCA and the coefficient of determination (r^2^) with the first difference FCA in the combined area of NBP, WGB and EGB. Only 2 variables have r^2^ values that are significant (positive) at p<0.1 (bold) and none are significant at p<0.05.

| **Variable** | **r^2^ with FCA** |
| --- | --- |
| Number of days with SST above 14˚C, July-August  Sum of daily SSTs above 14˚C, July-August  Average SST, July-August  Number of days with SST above 17˚C, July-August  Sum of daily SSTs above 17˚C, July-August  Temperature for 0-15 m at BY15, annual  Temperature for 0-15 m at BY15, Jun 24 - Sep 8  Temperature for 0-15 m at BY15, Jul 9 - Aug 24  Temperature for 0-15 m for NBP, annual  Temperature for 0-15 m for NBP, May 31 - Sep 4  Temperature for 0-15 m for NBP, Jul 2 - Sep 4  Temperature for 0-15 m Baltic Proper, annual  Temperature for 0-15 m in Baltic Proper, May 31 - Sep 4  Temperature for 0-15 m in Baltic Proper, Jul 2 - Sep 4  Sunshine duration, July-August  Shortwave direct irradiance, July-August  Shortwave irradiance, July-August  Eastward wind velocity, July-August  Northward wind velocity, July-August  Hypoxic area in the Baltic Proper, km^2^  Dissolved inorganic nitrogen (DIN)  Dissolved inorganic phosphorus (DIP)  Ratio DIN/DIP  Phosphorus excess  Phosphate concentration 0-15 m at BY15, May 25 - June 9  Phosphate concentration 0-15 m at BY15, June 9 - July 9  Salinity for 0-15 m at BY15, annual  Salinity for 0-15 m at BY15, May 25 - September 9  Salinity for 0-15 m at BY15, July 9 - August 24 | 0.043  **0.098**  0.094  0.050  **0.107**  0.000  0.029  0.061  0.000  0.009  0.012  0.001  0.011  0.014  0.037  0.049  0.035  0.035  0.015  0.002  0.006  0.004  0.000  0.001  0.001  0.000  0.000  0.002  0.001 |

Supplementary Table S2. Time series of FCA and their first differences in the Northern Baltic Proper (NBP), Western Gotland basin (WGB), Eastern Gotland Basin (EGB) and for the combined region (NBP+WGB+EGB). The first difference values (NBP', WGB', EGB' and (NBP+WGB+EGB)’) are derived by subtracting the last year's FCA value. For locations see Fig. 2.

| Year | NBP | WGB | EGB | NBP' | WGB' | EGB' | (NBP+WGB+EGB) | (NBP+WGB+EGB)' |
| --- | --- | --- | --- | --- | --- | --- | --- | --- |
| 1979 | 16.4 | 12.7 | 12.5 | NA | NA | NA | 14.6 | NA |
| 1980 | 10.2 | 12.4 | 3.3 | -6.2 | -0.3 | -9.2 | 8.5 | -6.1 |
| 1981 | 15.8 | 21.2 | 24.4 | 5.6 | 8.8 | 21.2 | 21.1 | 12.6 |
| 1982 | 1.7 | 12.7 | 11.7 | -14.1 | -8.5 | -12.8 | 9.3 | -11.8 |
| 1983 | 18.9 | 17.9 | 20.8 | 17.2 | 5.2 | 9.1 | 20.0 | 10.7 |
| 1984 | 16.8 | 13.8 | 7.0 | -2.1 | -4.1 | -13.8 | 13.3 | -6.7 |
| 1985 | 0.1 | 2.4 | 1.5 | -16.7 | -11.4 | -5.5 | 1.4 | -11.9 |
| 1986 | 4.9 | 7.7 | 2.7 | 4.9 | 5.3 | 1.2 | 5.1 | 3.7 |
| 1987 | 0.9 | 0.0 | 0.3 | -4.1 | -7.7 | -2.4 | 0.6 | -4.5 |
| 1988 | 5.1 | 2.5 | 3.3 | 4.2 | 2.5 | 3.0 | 4.1 | 3.6 |
| 1989 | 9.7 | 5.1 | 2.8 | 4.7 | 2.6 | -0.5 | 6.9 | 2.7 |
| 1990 | 5.3 | 4.9 | 1.6 | -4.5 | -0.2 | -1.2 | 4.1 | -2.7 |
| 1991 | 17.6 | 13.1 | 19.6 | 12.3 | 8.1 | 18.0 | 18.3 | 14.1 |
| 1992 | 29.3 | 8.6 | 17.9 | 11.6 | -4.4 | -1.7 | 21.5 | 3.3 |
| 1993 | 9.2 | 21.5 | 6.7 | -20.1 | 12.8 | -11.2 | 12.2 | -9.4 |
| 1994 | 21.4 | 22.1 | 24.4 | 12.3 | 0.6 | 17.7 | 23.8 | 11.7 |
| 1995 | 3.9 | 3.6 | 5.1 | -17.5 | -18.4 | -19.3 | 4.5 | -19.3 |
| 1996 | 4.7 | 2.1 | 3.1 | 0.7 | -1.5 | -2.0 | 3.6 | -0.9 |
| 1997 | 31.6 | 25.0 | 11.2 | 26.9 | 22.9 | 8.1 | 23.0 | 19.4 |
| 1998 | 4.1 | 3.5 | 7.7 | -27.5 | -21.5 | -3.4 | 5.7 | -17.3 |
| 1999 | 18.7 | 13.5 | 37.3 | 14.6 | 10.0 | 29.6 | 26.0 | 20.3 |
| 2000 | 30.0 | 28.3 | 25.7 | 11.2 | 14.9 | -11.6 | 20.9 | -5.2 |
| 2001 | 4.9 | 8.0 | 4.7 | -25.0 | -20.3 | -21.0 | 5.6 | -15.3 |
| 2002 | 33.5 | 15.6 | 17.6 | 28.6 | 7.6 | 12.9 | 23.8 | 18.2 |
| 2003 | 24.6 | 28.4 | 41.9 | -8.9 | 12.8 | 24.3 | 33.7 | 9.8 |
| 2004 | 4.4 | 9.3 | 9.7 | -20.2 | -19.2 | -32.2 | 7.7 | -25.9 |
| 2005 | 39.9 | 35.7 | 44.8 | 35.6 | 26.4 | 35.1 | 42.4 | 34.7 |
| 2006 | 4.5 | 9.8 | 14.0 | -35.4 | -25.8 | -30.8 | 9.9 | -32.5 |
| 2007 | 17.8 | 2.2 | 7.2 | 13.3 | -7.6 | -6.8 | 10.0 | 0.2 |
| 2008 | 30.7 | 36.9 | 47.2 | 12.9 | 34.7 | 39.9 | 40.3 | 30.3 |
| 2009 | 6.5 | 7.7 | 5.7 | -24.3 | -29.2 | -41.4 | 6.7 | -33.6 |
| 2010 | 15.7 | 5.0 | 11.0 | 9.2 | -2.6 | 5.3 | 11.9 | 5.1 |
| 2011 | 39.7 | 19.6 | 18.6 | 24.0 | 14.6 | 7.6 | 27.8 | 16.0 |
| 2012 | 12.1 | 13.4 | 11.0 | -27.6 | -6.2 | -7.6 | 12.4 | -15.4 |
| 2013 | 11.9 | 6.5 | 6.0 | -0.2 | -6.9 | -5.0 | 8.4 | -4.0 |
| 2014 | 49.6 | 21.8 | 43.5 | 37.7 | 15.3 | 37.5 | 42.1 | 33.7 |
| 2015 | 35.8 | 25.6 | 19.2 | -13.8 | 3.9 | -24.3 | 27.6 | -14.5 |
| 2016 | 24.8 | 8.5 | 14.3 | -11.0 | -17.1 | -4.9 | 17.6 | -10.1 |
| 2017 | 44.6 | 15.2 | 36.6 | 19.9 | 6.7 | 22.3 | 36.4 | 18.8 |

References

Kothe, S. *et al*. A satellite-based sunshine duration climate data record for Europe and Africa. *Remote Sens*. **9**, 429, doi:10.3390/rs9050429 (2017).

Mueller, R. *et al*. A new algorithm for the satellite-based retrieval of solar surface irradiance in spectral bands. *Remote Sens*. **4**, 622-647 (2012).

Mueller, R. *et al*. Digging the METEOSAT treasure - 3 decades of solar surface radiation. *Remote Sens*. **7**, 8067-8101 (2015).

Reynolds, R. W. *et al*. Daily high-resolution blended analyses for sea surface temperature. *J. Climate*, **20**, 5473-5496 (2007).

Savchuk, O.P. in *Chemical structure of pelagic redox interfaces: observation and modelling*: Large-scale dynamics of hypoxia in the Baltic Sea (ed. Yakushev, E.) 137-160 (Springer, Berlin, 2010).

Zhang, H.-M., Bates, J. J. & Reynolds, R. W. Assessment of composite global sampling: Sea surface wind speed. *Geophys. Res. Lett*., **33**, L17714, <http://dx.doi.org/10.1029/2006GL027086> (2006).
